# An Evaluation of Laboratory Efficiency in Shanghai Emergency by Turn Around Times Level

**DOI:** 10.1002/jcla.21775

**Published:** 2014-08-17

**Authors:** Yiming Lu, Waiian Leong, Bohua Wei, Ping Yu, Cuicui Wang, Yilin Ying, Tingsong Wang, Jianjing Tong, Dingliang Zhu, Jing Ye

**Affiliations:** ^1^ Emergency Department Shanghai Ruijin Hospital Shanghai Jiaotong University School of Medicine Shanghai China; ^2^ Joint laboratory of Vascular Biology of Health Science Center and Shanghai Institute of Hypertension Ruijin Hospital Shanghai JiaoTong University School of Medicine Shanghai China

**Keywords:** Shanghai Emergency, laboratory efficiency, turnaround times, point‐of‐care

## Abstract

**Objective:**

China launched a health care reform policy due to the aging population and rapid urbanization. However, emergency overcrowding is not improved. We assessed the laboratory efficiency of emergency department (ED) in Shanghai hospitals.

**Methods:**

We recorded the turn around times for processing laboratory biomarkers to assess laboratory efficiency at 17 EDs in national/regional hospitals. We compared TAT between national and regional hospitals and between central and ED laboratories to analyze the relationship between the laboratory efficiency and the ED overcrowding.

**Results:**

All the participating hospitals have an emergency laboratory. The median TAT for c‐TNT was 61 min (46–76 min) at regional EDs compared with 64 min (46–87 min) at national EDs; therefore, the TAT at regional EDs were more efficient (*P* < 0.05). The TAT were longer (65 min (53–85 min)) at ED labs than (60 min (42–83 min)) at central labs (*P* < 0.05), independent of the hospital tier and working period. We discovered that only 9% of investigated samples at Tier II EDs and 5% at Tier III EDs were assayed by point‐of‐care (POC) instruments.

**Conclusion:**

Our TAT level is approaching the recommended international standard. However, the TAT evaluation from ED laboratories demonstrates that their existence does not decrease the waiting time for laboratory reports compared to central laboratory. Thus, they have not yet approached a level to share the burden of the ED overcrowding. Further arrangement should be assigned to separate the function of emergency laboratory and central laboratory. It is worth deploying the POC assay in the ED, which will save twice the TAT level. The idea of evaluating routine laboratory efficiency by TAT at ED is fast, convenient, although it does not represent the general level of laboratory efficiency.

## INTRODUCTION

The Chinese government launched a reform plan in 2009, which included spending approximately US$125 billion to fund a public health provision 1. With this funding for primary public health care, the equipment at medical facilities, infrastructure development, and training sessions have been innovated. The Chinese medical environment is expected to gradually improve 2. However, Emergency department (ED) overcrowding is always one of the most severe problems facing the Chinese medical care system. We have investigated the potential causes that lead to ED overcrowding according to an analysis of patient triage in the ED of a well‐known representative hospital with overcrowding. Our previous retrospective study demonstrated that the ED overcrowding in the hospital is not simply due to the deficiency of infrastructure and medical supplies, because the department we interviewed have benefited from a government's health care reform 3, 4 and their medical equipment is currently improved among the best in the world 5.

In this study, we questioned whether the ED overcrowding is due to the low laboratory efficiency. Although an emergency laboratory has been reconstructed to relieve the pressure of central laboratory and to improve the laboratory efficiency at emergency, we had no idea whether the ED laboratory functions as estimated because there was no statistical prereform report. We then designed this study to evaluate the turnaround time (TAT) of routine biomarkers. We expected to know whether TAT from emergency laboratory is faster than those from central laboratory. We also compared the postreform laboratory efficiency to the international level of TAT. If it is approaching an international level, it will not only suggest an achievement of the reform, but also indicate that laboratory efficiency is not a prominent factor that leads to the particular severe overcrowding in Shanghai.

## MATERIALS AND METHODS

### Setting and Study Design

The study was designed to evaluate the laboratory efficiency. Based on the principle of “large samples and multiple center study,” we selected all tier III hospitals in 10 districts of Shanghai, which is the largest city in China with more than 24 million residents, and one representative tier II hospital in the district. In total, there were 7 tier III hospitals and 10 tier II hospitals in the group. All the enrolled hospitals constructed a lab in their EDs, which were defined as sectional laboratories for ED routine tests. Samples were randomly distributed by laboratory personnel to either a central lab at an inpatient hospital or to an ED lab. Emergency physicians recruited chest pain patients for the study when cardiac biomarkers were required for a diagnosis. A short training session was presented to the enrolled emergency physicians and laboratory personnel after the study was initiated in January 2011. The study lasted 8 months, spanning from April 2011 to December 2011.

### The Selection of Participants and Data Collection and Processing

The TAT was defined as the time point when the cardiac biomarker tests were ordered until the time point when the ED physicians were available to analyze the test results. The emergency physician completed a questionnaire (see Table 1), when they received the laboratory reports. The cardiac biomarkers tested included serum c‐TNT, CK‐MB, and myoglobin. All these laboratories we selected used same machine (Immunoassay system of BACKMAN and SYSMEX Corporation) and kits (Cat.: 331869, 560166, and 28165215) to detect the cardiac biomarkers in the study. By completing detailed questionnaires, the emergency physicians provided the following information regarding the tests: (1) the TAT values of cardiac biomarkers that were obtained in the ED using conventional or POC instruments; (2) whether the tests were performed in an ED or central laboratory; and (3) the types of instruments that were used to obtain the biomarker values.

**Table 1:** Characteristics of Survey Respondents

			Age			TAT of c‐TNT			TAT of CK‐MB			TAT of myoglobin		
		Overall(*n*)	*n*	y (mean (SD))	*P* value	*n*	min (P_50_ (P_25_,P_75_))	*P* value	*n*	min (P_50_ (P_25_,P_75_))	*P* value	*n*	min (P_50_ (P_25_,P_75_))	*P* value
Tier	II Hospital	410	402	69.94 (13.36)	0.8967	392	61 (46, 76)	0.0340	380	61 (49, 76)	0.0146	239	63 (47, 77)	0.0762
	III Hospital	501	487	70.37 (13.58)		498	64 (46, 87)		396	65 (47, 91.5)		313	65 (47, 91)	
Laboratory	Central	363	350	69.29 (13.13)	0.0739	363	60 (42, 83)	0.0050	281	58 (40, 81)	0.0001	215	57 (40, 80)	<0.0001
	Emergency	487	479	70.68 (13.72)		467	65 (53, 85)		449	65 (55, 83)		308	69 (56, 85)	
Period	Working	409	399	70.86 (13.26)	0.2241	401	64 (50, 83)	0.0006	359	65 (54, 85)	0.0006	246	66 (53, 83)	0.0052
	Night	473	463	69.48 (13.51)		460	60 (40, 78)		411	60 (42, 79)		301	60 (42, 80)	

### Primary Data Analysis

The study was designed as a retrospective and multicentre analysis. It focused on fast clinical decisions regarding acute chest pain in patients with a high risk of AMI or ACS. This observational investigation was aimed at detecting whether the TAT reach international standards and whether there is a possibility to alleviate the ED overcrowding by further improving the laboratory efficiency.

We used the Wilcoxon rank‐sum test and Kruskal‐Wallis tests to assess the differences between Tier II and Tier III hospitals and between central and ED laboratories if the results of the study failed to satisfy normal distributions (SAS procedures PROC UNIVARIATE and PROC NPAR1WAY). We considered a two‐sided *P*‐value of 0.05 or less statistically significant. From the aggregate data, we determined the TAT for the troponin, CK‐MB, and myoglobin tests. All the discrete data, including the TAT for c‐TNT, CK‐MB and myoglobin, are presented as the median (the quartiles). All the analyses were conducted using SAS software version 8.2 (SAS Institute Inc.).

### Role of the Funding Source

The sponsor of this study had no role in the study design, the data collection, the data analysis, the data interpretation, or the writing of the report. The corresponding author had full access to all the data in the study and made the final decision to submit the manuscript for publication. All the funding sources were used for the data collection, the statistical analysis and service fees.

## RESULTS

### 1. The Distribution of the Sample Collection and the Characteristics of the Survey Respondents

All 17 participating hospitals we selected have constructed or expanded ED laboratories due to the medical reform policy with the financial support of the health care reform plan. Therefore, we are sure the TAT levels we recorded are not influenced by the limitation of the infrastructure or medical supply. Among these hospitals, 7 were Tier III, and 10 were Tier II. A total of 911 individuals were interviewed and completed questionnaires. Among the 911 questionnaires, 410 were from Tier II hospitals and 501 were from Tier III hospitals. The patient baseline characteristics were similar between groups (*P* > 0.05, see Table 1).

**Figure 1:**
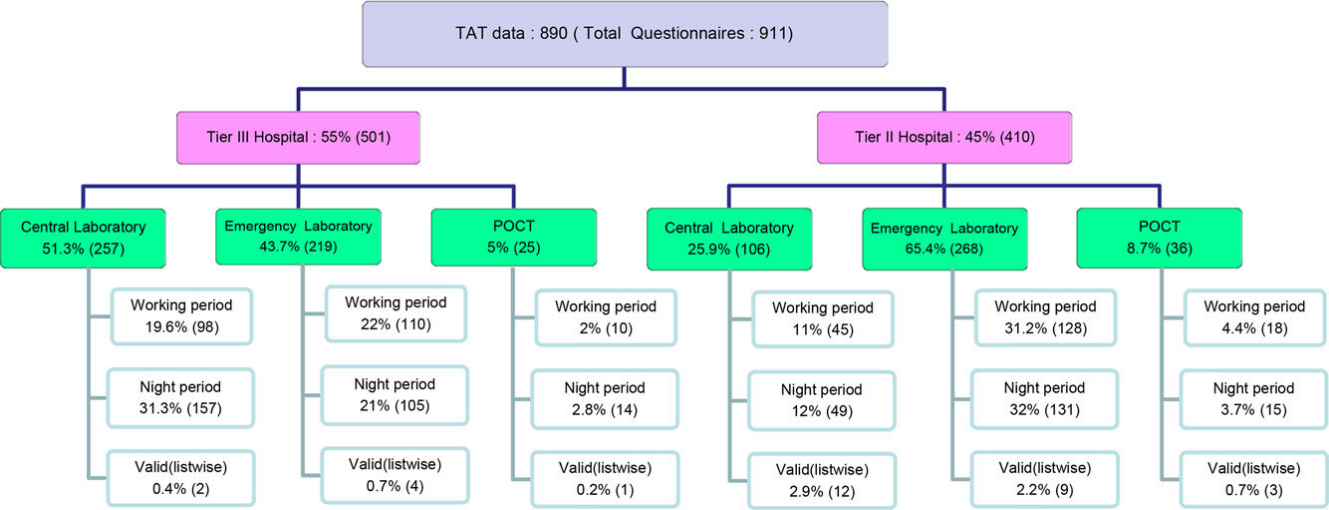
The distribution of sample collection. The case number of collected samples in each group is indicated in the brackets, and the percentage is calculated by the case number in the brackets divided to the upgrade case number.

We chose TAT of cardiac biomarkers from patients who were suffering from chest pain to evaluate the ED clinical efficiency, because chest pain is one of the most dangerous and representative symptoms in the ED physician. The distribution of the sample collection and the characteristics of the survey respondents are shown in Figure 1 and in Table 1. Of all the returned questionnaires, 890 (97.7%) reported TAT for c‐TNT, 776 (85.2%) reported TAT for CK‐MB and 552 (60.6%) reported TAT for myoglobin.

The clinicians and the laboratory personnel work toward a TAT goal of <60 min for cardiac biomarkers 6, 7; however, our results demonstrated that some of the EDs in the Shanghai hospitals did not meet the ideal TAT after the health care reform policy was implemented. To elucidate the reasons that led to prolonged TAT, we further analyzed the TAT among different groups. The results of c‐TNT assays are more crucial than those of other cardiac biomarker tests and c‐TNT results were reported more often (97.7%) than the other cardiac biomarkers; therefore, we selected the c‐TNT TAT for further analysis as representative data of laboratory efficiency.

### 2. The Assessment of Laboratory Efficiency at Central and ED Laboratories According to the TAT

**Figure 2:**
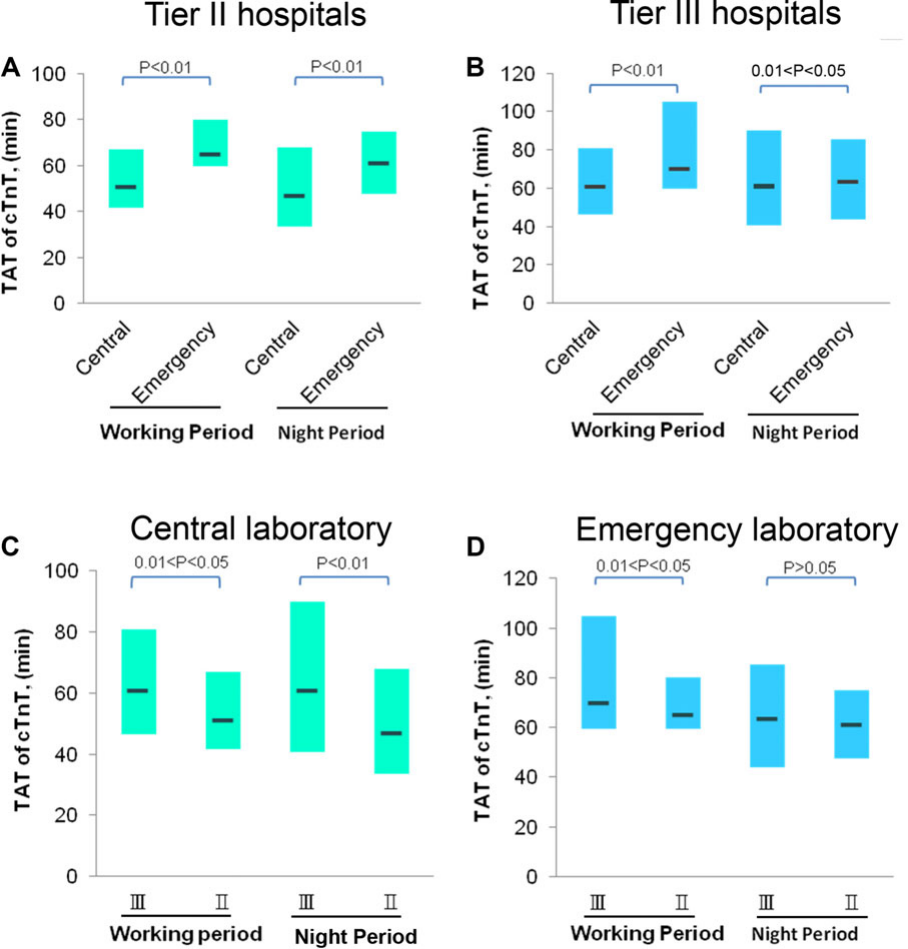
Analysis of TAT values between tier II and tier III hospitals, as well as between Emergency and Central laboratory. *P* values are indicated. (A) TAT P50 (P25, P75) of c‐TNT at working (8:00 to 17:00) and night period （17:01 to 7:59） from Central or Emergency Laboratory in tier II Hospital. (B) TAT P50 (P25, P75) of c‐TNT at working and night period from Central or Emergency Laboratory in tier III Hospital. (C) TAT P50 (P25, P75) of c‐TNT at working and night period from Central laboratory of tier II and tier III hospitals. (D) TAT P50 (P25, P75) of c‐TNT at working and night period in Emergency laboratory of tier II or III hospitals.

ED labs were constructed to decrease the waiting times for laboratory reports and prevent patient congestion in observation units and emergency clinics. To assess the efficiency of ED labs, we analyzed the TAT for cardiac biomarkers from central labs and ED labs. Surprisingly, the TAT from ED labs were longer than those from central labs (*P* < 0.05), which contradicts the purpose of creating an ED lab (see Table 1). To determine whether the hours of operation affected the TAT at central and ED labs, we divided the collection data according to working period (8:00–17:00) and night period (17:01–7:59). The median (P_25_, P_75_) TAT for c‐TNT between central and ED labs were 61 min (47–81 min) versus 70 min (60–105 min) in Tier III (*P* < 0.05), and 51 min (42–67 min) versus 65 min (60–80 min) in Tier II (*P* < 0.05) in working period. In night period, the median (P_25_, P_75_) TAT is 61 min (41–90 min) versus 63.5 min (44.5–85.5 min) (*P* < 0.05), and 47 min (34–68 min) versus 61 min (48–75 min) (*p* < 0.05). The TAT for c‐TNT at the ED labs in Tier II and Tier III hospitals were significantly longer than those at central labs *(P* < 0.05), regardless of the hours of operation (see Fig. 2, Table 1).

**Figure 3:**
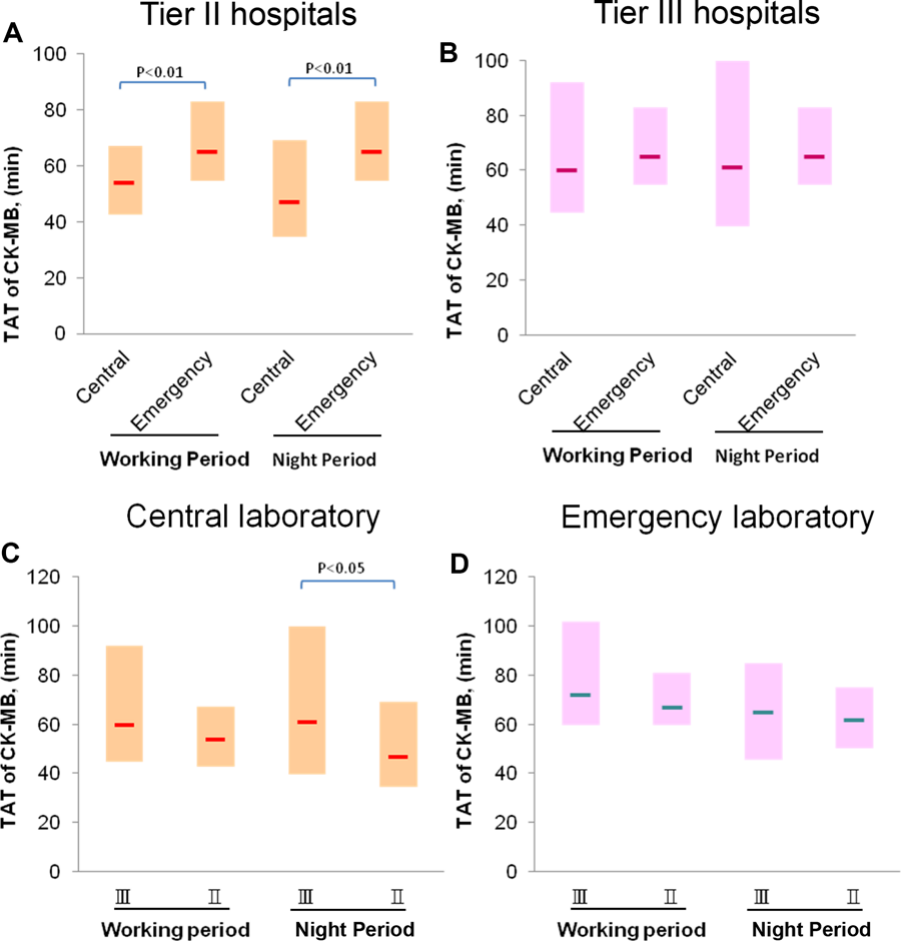
Analysis of TAT values between tier II and tier III hospitals, as well as between Emergency and Central laboratory. *P*‐values are indicated. (A), (B), (C), (D) TAT P50 (P25, P75) of CK‐MB as shown before Figure.

**Figure 4:**
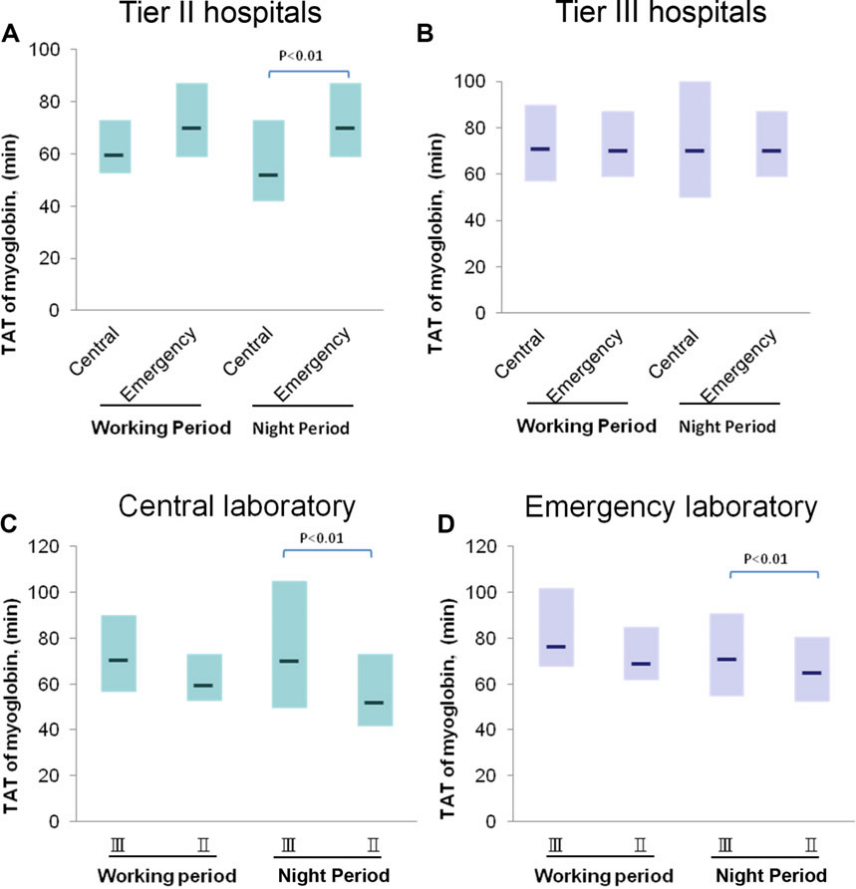
Analysis of TAT values between tier II and tier III hospitals, as well as between Emergency and Central laboratory. *P* values are indicated. (A), (B), (C), (D) TAT P50 (P25, P75) of myoglobin as shown before Figure.

The median (P_25_, P_75_) TAT for CK‐MB between central and ED labs were 60 min (45–92 min) versus 65 min (55–83 min) in Tier III and 54 min (43–67 min) versus 65 min (55–83 min) in Tier II in working period; and there were 61 min (40–100 min) versus 65 min (55–83 min), and 47 min (35–69 min), versus 65 min (55–83 min) in night period, respectively. Moreover, the median (P_25_, P_75_) TAT for myoglobin were 70.5 min (57–90 min) versus 70 min (59–87 min) in tier III and 59.5 min (53–73 min) versus 70 min (59–87 min) in tier II in working period; as well as 70 min (50–105 min) versus 70 min (59–87 min) and 52 min (42–73 min) versus 70 min (59–87 min) in night period. The TAT for CK‐MB and myoglobin were also longer at the ED laboratories (see Figs. 3 and 4, Table 1), but this is not statistically significant. This might be due to the fact there were less requirement for these two cardiac biomarkers and less reported data have been collected compared to the c‐TNT. These findings indicate that ED labs do not function more efficiently than central labs according to the TAT for measuring cardiac biomarkers, which is out of previous subjective inference and expectation. These results also suggest if we improve the laboratory efficiency of ED laboratories, it may relieve patient flow and alleviate ED congestion.

### 3. The Assessment of Laboratory Efficiency at Tier II and Tier III EDs According to the TAT

The situation of Chinese ED overcrowding in Tier III hospitals are more stressful than in tier II hospital 5. If the ED laboratory efficiency really plays an important role in the ED overcrowding crisis, it should be less efficient in tier III hospitals than in tier II hospitals. To further elucidate the question, we assessed the TAT between Tier II and Tier III hospitals. Indeed, our results demonstrate that the TAT for c‐TNT at Tier II hospitals were significantly shorter at tier III hospitals (*P* < 0.05, see Fig. 2). The median (P_25_, P_75_) TAT for c‐TNT was 70 min (60–105 min) at Tier III EDs compared with 65 min (60–80 min) at Tier II EDs; in addition, there was 61 min (47–81 min) compared to 51 min (42–67 min) at central labs in working period. In night period, the median (P_25_, P_75_) TAT is 63.5 min (44.5–85.5 min) versus 61 min (48–75 min) (*p* > 0.05), and 61 min (41–90 min) versus 47 min (34–68 min) (*P* < 0.05). The TAT for CK‐MB and myoglobin were also shorter at Tier II hospitals. The median (P_25_, P_75_) TAT for CK‐MB were 61 min (40–100 min) at Tier III central laboratories versus 47 min (35–69 min) at tier II in night period *(p* < 0.05, see Fig. 3C and Table 1).

Moreover, the median (P_25_, P_75_) TAT for myoglobin were 70 min (50–105 min) at tier III central labs versus 52 min (42–73 min) at Tier II; and 71 min (55–91 min) versus 65 min (52.5–80.5 min) at ED laboratories in night period *(P* < 0.01, see Fig. 4 and Table 1). This finding was further confirmed in the analysis of the independent TAT in the central and ED labs in these two types of hospitals, which indicates that the reporting of routine laboratory results, such as cardiac biomarkers is more efficient in Tier II hospitals.

These data contribute to the explanation why the ED in tier III hospitals have more overcrowding crisis than the tier II hospitals, although we have no evidence to indicate the direct strong relationship between TAT and overcrowding crisis. It confirms our hypothesis that long TAT, together with the low laboratory efficiency are one of the causes that lead to the ED overcrowding in Shanghai.

Moreover, the TAT level in tier III emergency laboratory is more variable than in tier II hospital, further indicating that the laboratory efficiency in tier III emergency is not stable or overloaded due to the flow of patients (Figs. 2, 3, 4). These data will encourage and persuade patients to visit tier II hospitals instead of rushing to tier III hospitals for some certain point.

### 4. Current POC Panel Usage in EDs After Health Care Reform

**Figure 5:**
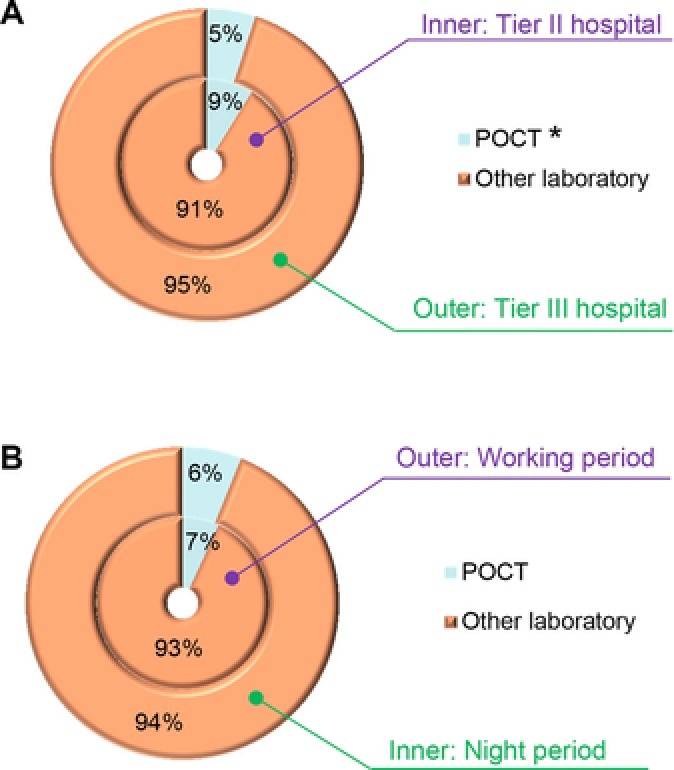
Analysis of the percentage of the samples assayed by POC panels. (A) The percentage of application of POC panels in Tier II or III hospitals. (B) The percentage of the application of POC panels in Working and Night period (*P < 0.05).

Another peculiar phenomenon that we discovered in this study was that point‐of‐care (POC) cardiac biomarker panels, which are widely used in the United States (US) and Europe, were not well recognised in Shanghai. However, we discovered that only 9% of samples at Tier II EDs and 5% of samples at Tier III EDs from the investigated patients were assayed by POC instruments (Fig. 5A). In addition, there were 7% of samples in working period and 6% of samples in night period (Fig. 5B). Since the international standard of POC assay were proposed at 30 min in the guidelines 8, 9, 10, 11, it will further decrease our TAT value and increase the ED laboratory efficiency twice than before, if our ED enhances the spread of POC assay and pursue the correct implication of POC assay.

## DISCUSSION

China has been experiencing a health care reform since 2009, and the Chinese medical environment is expected to gradually improve 1. However, ED overcrowding is not relieved. There are few statistical reports on analyzing the factors that affect Chinese overcrowding. We wondered that laboratory efficiency are considerably low in Chinese ED, and that might contribute to the ED overcrowding 12. We then examined the clinical efficiency of EDs by assessing the TAT for cardiac biomarkers because fast clinical decisions in the ED are mandatory to improve clinical efficiency. Indeed, we discovered that TAT in our EDs have certain specific features which is not seen elsewhere in the world and lead to the laboratory inefficiency.

### 1. The Assessment of the TAT from Central and ED Laboratories Indicates that Sectional Laboratories in EDs do not Improve Laboratory Efficiency

Unexpectedly, the TAT at ED laboratories were longer than those at central laboratories (see Table 1 and Figs. 2, 3, 4). Our experiment presents with solid data that ED laboratories do not act as an inevitable part for the emergency diagnosis as expected, although additional large‐scale and multicentre studies are required to compare the laboratory efficiency and elucidate whether this phenomenon is due to inappropriate administrative management of EDs. Other possible factors that affect the laboratory efficiency in the manuscript, including the management of the laboratory, the efficiency of laboratory staff, the protocol or kits used and the machine they used. All these hospitals we selected used same kits. Different methods increase the possibility of the variation of this study, but the expected TAT value suggested by the international guideline should be below 60s independent of methods and kits. Thus, we cannot exclude the hypothesis that the inefficiency of emergency laboratory is not due to the lack of the sufficient laboratory staff compared to the large amount of patient flow or due to the hospitals are more significant in night period further indicate the above hypothesis. In fact, the emergency at night‐shift is not managed reasonably, because the peak flow for emergency patients is due to the out‐clinic patients who miss the clinics and rush to the emergency at this moment. Thus, we propose that the administrative strategy must be pursued to increase the staff numbers at ED laboratories to ensure the laboratory efficiency or to strictly follow the emergency indications. This might be a solution to the fast diagnosis and treatment in the emergency and contribute to the alleviation of the ED overcrowding.

### 2. TAT Results Convince Patients to Avoid Overcrowding in Tier III Hospitals

This study presents with encouraging data that laboratory efficiency of routine laboratory assay is similar to regional and national hospitals. It is even more stable in regional hospitals. This will convince patients to choose regional hospitals, which will save time of routine emergent medical care with ensured medical quality and relieve patient overcrowding at the EDs in national hospitals.

### 3. Improvement of POC assay is a Significant Solution to Improve ED Laboratory Efficiency and Relieve Overcrowding

In this study, only 9% of samples at Tier II EDs and 5% of samples at Tier III EDs were assayed by POC panel (see Fig. 5). Such a low percentage of the application of the POC panel could be a direct cause that leads to the delay of emergency diagnosis and miss of the opportunity for the intervention therapy, etc. The POC assay can be limited to less than 30 min, twice less than TAT of laboratory assay 4, 6, 7, 8, 9. We will perform further study to analyze the reasons that inhibit the deployment of POC panel. One can imagine that it could be a significant solution to improve the laboratory efficiency and alleviate overcrowding in our ED by spreading the usage of POC panel and limit the POC assay under 30 min 13.

## LIMITATIONS

This study found encouraging data regarding the implementation of health care reform. The clinical efficiency of laboratory reporting is similar at regional and national hospitals. Patients tend to choose regional hospitals, which will relieve patient overcrowding in the EDs at national hospitals. This issue was one of the most challenging problems in Chinese health care before the reform plan was enacted.

The proper implementation of reform requires independent scientific assessments to enable midcourse corrections. Our study indicates that reform can fail if not implemented and modified properly. The results of the TAT evaluation of central and ED labs do not support the goal of creating an ED lab to increase clinical efficiency. Whether the health care reform should focus on constructing ED laboratories or central laboratories will depend on additional independent and evidence‐based studies.

The POC cardiac biomarker panels are widely used in the United States (US) and Europe. Therefore, we highly advocate improving the spreading usage and standard application of POC assay with the next version of the medical reform policy, which should be based on additional scientific studies and evaluations. These improvements will increase the clinical efficiency and cost‐effectiveness of EDs and will enhance Chinese medical reform after the reconstruction of a basic infrastructure.

Overall, our study of TAT evaluation demonstrated that ED laboratories have not yet approached a level to share the burden of patient flow at emergency department. Further arrangement should be assigned to separate the function of emergency laboratory and central laboratory. The idea of evaluating routine laboratory efficiency by TAT in the ED is fast, convenient, although it does not represent the general level of laboratory efficiency.
